# Resilience and entrepreneurial intentions of people with disabilities: in search of the Sustainable Development Goals (SDGs)

**DOI:** 10.12688/f1000research.113565.1

**Published:** 2022-07-01

**Authors:** Noemí Pérez-Macías, José L. Fernández-Fernández, Antonio Rúa Vieites

**Affiliations:** 1 Management, Universidad Pontificia Comillas, Madrid, 28015, Spain; 2 Management, Universidad Pontificia Comillas, Madrid, 2015, Spain; 3 Quantitative Methods, Universidad Pontificia Comillas, Madrid, 2015, Spain

**Keywords:** People with Disabilities, Resilience, Entrepreneurial Intentions, Entrepreneurship, Personal Factors, Perceived Collective Efficacy Sustainable Development Goals, Partial Least Squares-Structural Equation Modeling.

## Abstract

**Background: **There is a significant gap in employment between people with and without disabilities, despite the importance of work in achieving their independence, autonomy, and integration into society. There are several reasons that cause this gap to exist, such as: people with disabilities feel less prepared, there is a stigma or discrimination to hire people with disabilities and the incompatibility of schedules due to medical issues, among others. That is why entrepreneurship emerges as a good option for the integration of people with disabilities in our society, improves their confidence and promotes some of the Sustainable Development Goals set out in the 2030 Agenda. According to existing literature, people with disabilities have certain virtues such as resilience and motivation that favor entrepreneurship. Thus, the purpose of this study is to provide new insights into the variables that determine the entrepreneurial intention of people with disabilities.

**Methods:** In order to respond to this objective, an online questionnaire was given to people with disabilities between the ages of 16 and 65 years, residing in diverse regions of Spain. To analyze the results, this study uses Partial Least Squares-Structural Equation Modeling (PLS-SEM) in a sample of 235 people with disabilities in Spain using as a framework Krueger´s improved model, adding resilience as a new variable.

**Results: **The results reflect the importance of resilience, the subjective norm, and perceived collective efficacy in the entrepreneurial processes of people with disabilities.

**Conclusions:** This study contributes to the underdeveloped literature on entrepreneurship in people with disabilities; it provides insights that can have a practical effect on the reduction of the inequality gap between people with and without disabilities making recommendations to clinicians, vocational psychologists, and policymakers; also, this study would advance the achievement of Sustainable Development Goals 8 and 10.

## Introduction

According to the UK Equality Act 2010 “Persons with disabilities include those who have long-term physical, mental, intellectual or sensory impairments which in interaction with various barriers may hinder their full and effective participation in society on an equal basis with others”. In Spain, in fact, for personal income tax purposes and according to the ODISMET, ^1^ people with disabilities are considered disabled if they show a degree of disability (ranked from 0 to 100%) equal to or greater than 33%. For people with disabilities, work is a fundamental tool that increases social welfare and savings on social costs in addition to supporting autonomy, freedom, and independence for people with disabilities. ^2^^–^^4^ However, there is an important employment gap between people with and without disabilities, ^5^^,^^6^ and people with disabilities are less likely to find jobs. ^1^^,^^7^^,^^8^ On the one hand, this could be because people with disabilities may think they are not prepared for employment ^9^ and may depend on social assistance ^9^^,^^10^; on the other hand, they face certain social barriers to employment, such as prejudice, discrimination or marginalization. ^5^^–^^7^^,^^11^^–^^13^ This employment gap not only increases inequalities and social exclusion, but also undermines the Sustainable Development Goals (SDG) and Spain’s own national legislation.

Given the increasing number of unemployed people with disabilities, ^1^ it is worthwhile considering entrepreneurship ^14^ as an alternative means of income and development. This approach could increase the gross domestic product (GDP), ^3^^,^^5^^,^^7^^,^^8^^,^^12^^,^^13^^,^^15^^–^^18^ and improve economic development. ^6^ On an individual level, self-employment could improve their social integration and increase self-esteem for people with disabilities. Advancing the economic inclusion of people with disabilities would show progress toward the 2030 Agenda and some of the Sustainable Development Goals (SDGs), which expressly advocate for reduced inequality and peaceful societies that achieve sustainable development.

Entrepreneurship is a feasible option for people with disabilities, as it offers the possibility of accommodating their needs and establishing independence. ^19^ However, in Spain, compared with other countries such as USA, UK, or Australia, only 11.2% of people with disabilities are entrepreneurs, ^20^^–^^22^ which indicates under participation among this population. ^23^ In fact, the *Inclusive Entrepreneurship Policies: Country Assessment Notes* ^24^ report indicates that there is no specific legislation or specific action plan to promote entrepreneurship among people with disabilities. This statement is not surprising, since the General Law on the Rights of People with Disabilities and Social Inclusion (Real Decree 1/2013 from November 29th) clearly discusses the need to promote entrepreneurship in Spain.

It is important to identify the specific variables that foster entrepreneurship among people with disabilities, who, according to existing literature, have certain virtues such as resilience and motivation that favor entrepreneurship. ^2^^,^^7^^,^^13^^,^^18^^,^^25^^,^^26^

This article aims to deepen the knowledge about the variables that determine the EI-Entrepreneurial_Intention of people with disabilities. Since existing literature associates RES-Resilience with entrepreneurship, we adopt Krueger’s ^27^ improved model, as model framework including several modifications following Esfandiar *et al*. ^28^ and add the RES-Resilience construct. ^29^

As a practical contribution, we aim to achieve three objectives: improve social integration of people with disabilities, ^5^^,^^30^^,^^31^ reduce costs of public social services, ^4^ and propose ideal and efficient policies regarding social integration and entrepreneurship.

Furthermore, the immediate justification of this work stems from the fact that research on this subject is scarce, ^32^ especially in Spain. ^33^ Prior research suggests continued investigation, similar to the study presented in this article. ^6^^,^^7^^,^^13^^,^^16^^,^^25^^,^^30^^,^^31^^,^^34^

## Theoretical foundations

### Krueger’s ^27^ improved model and entrepreneurial intentions

**Figure 1.**
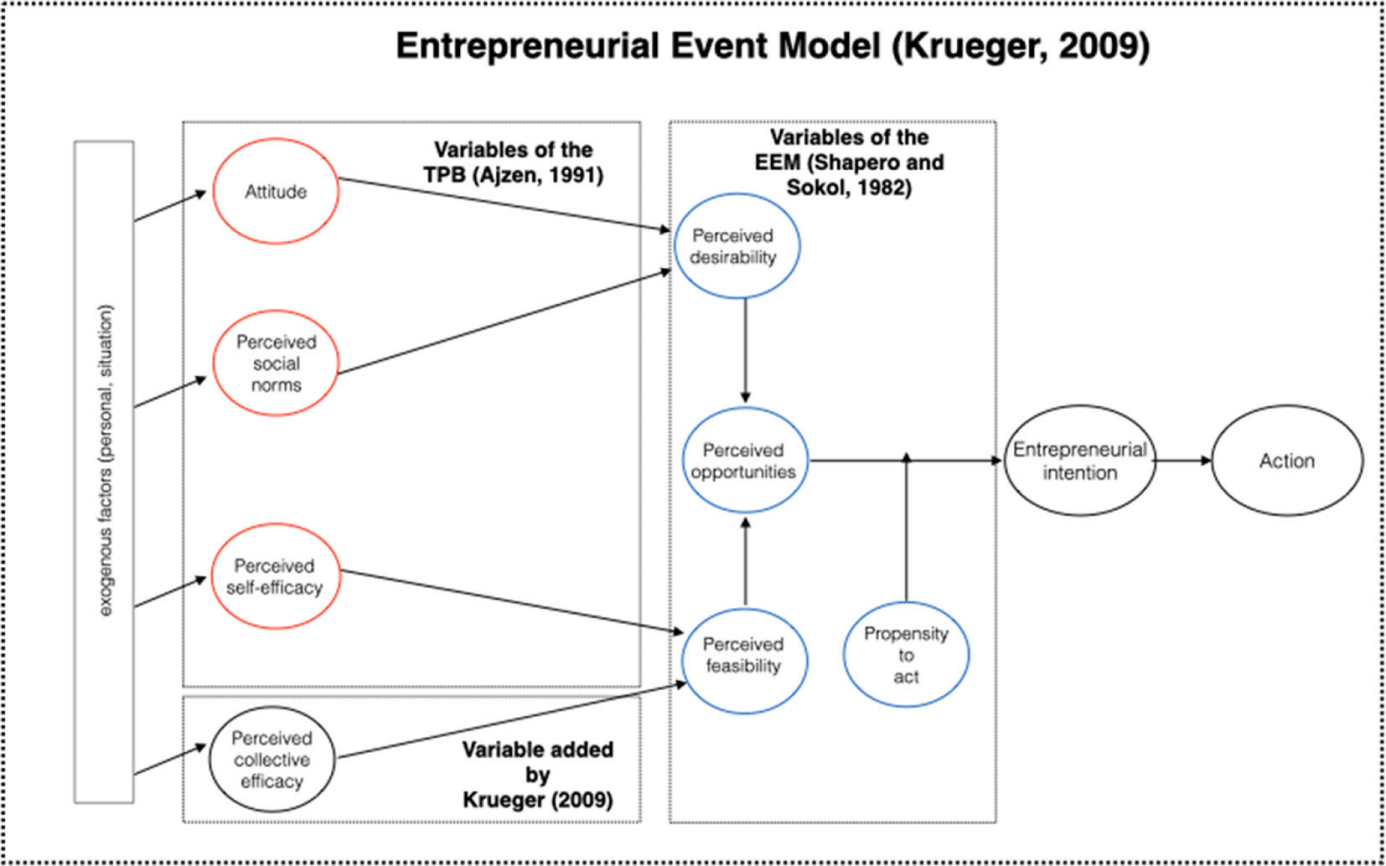
Intention Model. This figure has been adapted from Krueger ^27^ with permission from Springer Nature, by including the specifications about the models that were included in this figure. Krueger's Entrepreneurial Intention Model ^27^ is described. This model consists of attitude, perceived social norms that influence the perception of desirability. Perceived self-efficacy and perceived collective efficacy influence perceived feasibility. Both, perceived desirability and perceived feasibility influence perceived opportunity, which in turn influences entrepreneurial intention and is moderated by the propensity to act. Finally, entrepreneurial intention influences action.

Krueger ^27^ proposes a new model (see Figure 1) that improves the predictive capability of the frameworks of understanding entrepreneurial intention (EI) by integrating the theory of planned behaviour (TPB) and the entrepreneurial event model (EEM). This improvement to the model responds to the fact that, although TPB ^35^ is the most used theory in the entrepreneurship literature and one of the standards to predict entrepreneurial intentions (EI), it does not consider external influences. ^27^ According to TPB, there are three attitudes that precede EI, namely, the *attitudes towards the act* itself, in this case A_Attitudes_Towards_the_Behaviour –the individual’s assessment of their desire to create a new project; the SN_Subjective_Norm –or the perception one has about what the people around him/her think about becoming an entrepreneur; and the PBC_Perceived_Behavioural_Control *–*or the perception the individual has about the absence or presence of the resources and opportunities to develop a certain behaviour. ^35^

Kruger ^27^ states that the EEM ^36^ considers external factors but does not consider social norms. When this is considered, social norms add a predictive value to the entrepreneurial intentions (EI) model. This model establishes that the EI predictors are PD-Perceived_Desirability –the individual’s will to become an entrepreneur-, ^36^^,^^37^ PF_Perceived_Feasibility - the individual’s belief that he or she possesses the skills to be an entrepreneur-, ^36^ and the *propensity to act* –how an event can trigger a specific action-. Even though both models are similar, they are not identical. Each has its own specificities. ^38^^,^^39^

The resulting model proposed by Krueger ^27^ adds PCE_Perceived_Collective_Efficacy as an additional variable. In our case, this variable can be significant because many of these individuals depend on (or at least frequently request the participation of) others to perform some activities. ^6^^,^^40^

### Entrepreneurship among people with disabilities: Factors that influence EI

According to Abbas and Khair, ^23^ people with disabilities consider entrepreneurship feasible. This is partly because of the barriers they face in terms of employment. ^7^^,^^8^^,^^12^^,^^26^ In addition, they may have some personality traits that favor entrepreneurship, such as resilience, tolerance to uncertainty, and persistence. ^2^^,^^7^^,^^13^^,^^25^^,^^26^ Academic studies on EI analysis among people with disabilities are still scarce, especially in Spain. ^2^^,^^33^^,^^41^^–^^43^ This is despite the fact the advantages that entrepreneurship presents and the important social benefits it can bring. ^2^^,^^5^^,^^13^^,^^16^^,^^17^^,^^25^^,^^26^^,^^30^^,^^31^^,^^43^^,^^44^ Therefore, it is of interest to conduct studies that contribute to increasing the number of entrepreneurs among people with disabilities.

In this regard, Pérez-Macías and Fernández-Fernández ^2^ qualitatively approximate the factors that influence the EI of people with disabilities. They point out the following: First, they identify the cognitive, personal, and psychological factors, as well as motivators and personal barriers. They then consider factors related to entrepreneurship training. In the same way, they point out contextual factors (e.g., role models and social capital) and institutional factors, as well as barriers or environmental support. Even so, we must specify that the influence of the factors detailed on EI will depend on the context, as well as on possible bias among the sample.

Along with institutional and environmental factors, EIs are influenced by personal dynamics. ^45^^,^^46^ For example, there is evidence that people who face important challenges in life, such as an acquired disability, tend to develop greater RES_Resilience, as well as motivational, cognitive, and social resources that help them successfully adapt to the situation. ^47^ For some authors, this psychological mechanism boosts entrepreneurship among people with disabilities. ^7^ Given this context, we focus on these personal factors.

## Development of hypothesis

### Krueger's model as a framework of reference with the adaptations of Esfandiar et al.

**Figure 2.**
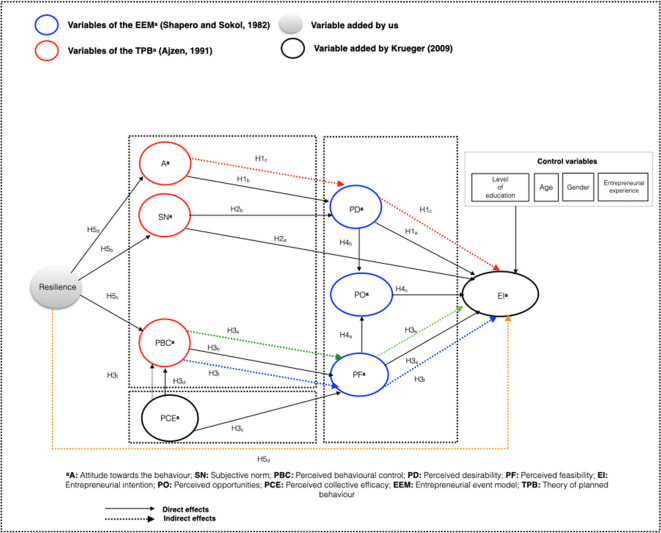
Robust model for predicting Entrepreneurial Intention. This figure shows our proposed model. We are establishing resilience direct influence on attitude, subjective_norms and perceived_behavioral_control (H5a;H5b;H5c). It is also indicated that it indirectly affects EI (yellow_dashed_line_H5d). It is established that attitude directly influences perceived_desirability (H1b) and indirectly influences EI (red_dashed_line_H1c). Subjective_norms directly affects perceived_desirability and EI (H2b;H2a). Perceived_behavioral_control directly affects perceived_feasibility (H3b) and indirectly affects EI (green_dashed_line_H2e). Perceived collective efficacy directly affects perceived_behavioral_control (H3d) and indirectly affects EI (blue_dashed_line_H3f). Perceived_desirability directly affects EI and perceived_opportunities (H1a; H4b). Perceived_factibility_perceived directly affects EI and perceived_opportunities (H3a;H4a). Finally, it is established that perceived_ opportunities directly influences EI (H4c).

We start with Krueger’s improved model ^27^ (see Figure 1), which has been validated in studies conducted on people who do not have disabilities. ^28^^,^^48^ In this study, we use an enriched version that incorporates *resilience* (see Figure 2), which other authors have identified as relevant in studies with people with disabilities. ^2^ The objective is to establish mechanisms that aid the rise of entrepreneurship among this group while reducing their vulnerabilities in the job market. The proposed model is illustrated in Figure 2.

Shapero and Sokol ^36^ prove that individuals that have a high PD-Perceived_Desirability had a greater probability of becoming entrepreneurs. This is corroborated by others who say that, when an individual perceives becoming an entrepreneur as desirable, it increases their EI. ^28^^,^^39^^,^^49^ At the same time, Shapero and Sokol ^36^ mention how cultural aspects, as well as family, can increase the PD-Perceived_Desirability to become an entrepreneur; in turn, they affect EI positively.

Therefore, people with disabilities who decide to become entrepreneurs must have previously built a favorable A-Attitude ^6^ when they identified entrepreneurship as a possible way to reach the levels of freedom and independence they desire. ^3^^,^^5^^,^^16^ Furthermore, they may believe that financial self-reliance will remove some of the barriers that restrict their full integration into the community. ^17^^,^^22^^,^^48^ Together, these factors contribute to increased EI whenever entrepreneurship is considered a desirable option. Shook and Bratinau ^49^ concur and articulate that the more favorable the individual’s efforts towards becoming an entrepreneur, the higher their PD-Perceived_Desirability. Consequently, we developed the following hypotheses:

*H1 _a_: PD-Perceived_Desirability has a direct effect on EI.*

*H1 _b_: A-Attitude_towards_Entrepreneurship has a direct effect on PD-Perceived_Desirability*

*H1 _c_: A-Attitude_towards_Entrepreneurship has a direct effect on EI.*

The SN-Subjective_Norm, which, in general terms, can influence certain behaviors and opinions that, in turn, affect EI, ^35^ refers to the way individuals interpret the opinions of their significant others and how they can affect their own likelihood of becoming entrepreneurs. ^50^ In fact, in the case of people with disabilities, family members and friends have a strong influence on their decision making, ^6^ extending to EI itself. However, existing studies are not conclusive regarding the degree to which this is true. In fact, the influence of close relations is one of the variables that generates controversy in entrepreneurship studies of people without disabilities. Some studies state that it has a direct influence on EI, ^51^ whereas others say it is indirect, through PBC-Perceived_Behavioural_Control and A-Attitude_Towards_Entrepreneurship, ^52^ or through PF-Perceived_Feasibility and PD *-*Perceived_Desirability. ^48^ Other studies have concluded that there is no influence at all. ^28^^,^^53^

For our part, we developed the hypothesis that the SN-Subjective_Norm exerts a direct influence, either over the EI of people with disabilities or their PD-Perceived_Desirability. This is in line with other authors ^39^ that show how parental support, because of their economic and emotional dependence, has a strong influence on the PD-Perceived_Desirability of college students. Similarly, Solesvik *et al*. ^54^ state that family bonds and other cultural aspects can influence individuals’ PD *-*Perceived_Desirability towards entrepreneurship and EI. Therefore, we consider the opinions of family members, friends, and classmates about PD-Perceived_Desirability and EI to be significant among people with disabilities, who are influenced either at an emotional or economic level. ^6^^,^^13^ In this sense, explicit support will make entrepreneurship desirable. ^55^ Consequently, we propose the following hypotheses:

*H2 _a_: SN-Subjective_Norm has a direct effect on EI.*

*H2 _b_: SN-Subjective_Norm has a direct effect on PD-Perceived_Desirability.*

People that normalize adapting to change, as many people with disabilities, ^13^ must develop certain psychological resources that allow them to spot business opportunities, better confront uncertainty, mobilize resources that improve their ability to succeed in new circumstances, and focus on a specific career goal. ^56^ Such resources tend to favor PF-Perceived_Feasibility because of the development of capabilities, such as persistence, resilience, independent problem-solving, creativity, and innovation. ^13^^,^^57^ These characteristics are related to successful entrepreneurship. ^58^

PBC-Perceived_Behavioural_Control plays an important role in entrepreneurship, ^59^ even in the face of adversity. ^58^ Thus, it has been said that people who struggle and survive the difficulties that a disability brings can develop a higher level of optimism and higher risk tolerance. These two traits could, likewise, increase the ability to overcome challenges ^60^ and their PBC-Perceived_Behavioural_Control. Ultimately, this could increase the PF-Perceived_Feasibility and reinforce EI.

By contrast, given the challenge that people with disabilities have in finding employment, they often develop a higher level of persistence and improve creative problem-solving skills. ^13^ These skills increase PBC-Perceived_Behavioural_Control by supporting the development of a greater PF-Perceived_Feasibility, which can be translated into higher EI. ^7^ In fact, there is evidence that people with disabilities who develop strong *self-efficacy* or PBC-Perceived_Behavioural_Control and a higher level of PF-Perceived_Feasibility manage to influence their EI significantly. ^16^

Overall, although the support of family members and friends is a required condition, ^2^^,^^13^ it is not enough to become an entrepreneur. ^6^^,^^61^ Hence, those who become entrepreneurs may need the support of others to help develop new networks ^13^ and access new funding and resources. This support will result in a reinforcement of the PCE-Perceived_Collective_Efficacy, ^6^ which could increase individual PBC-Perceived_Behavioural_Control regarding a particular action, such as entrepreneurship. ^62^ Additionally, it can also increase PF-Perceived_Feasibility which in turn could increase EI. ^28^ This appears to be because when an individual thinks that their support system (e.g., friends or co-workers) can help them successfully address any problems that could arise, then it is more likely that they will decide to become an entrepreneur. ^62^ Given all the above-mentioned considerations, we propose the following hypotheses:

*H3 _a_: PF-Perceived_Feasibility has a direct effect on EI.*

*H3 _b_: PBC-Perceived_Behavioural_Control has a direct effect on PF-Perceived_Feasibility.*

*H3 _c_: PCE-Perceived_Collective_Efficacy has a direct effect on PF-Perceived_Feasibility.*

*H3 _d_: PCE-Perceived_Collective_Efficacy has a direct effect on PBC.*

*H3 _e_: PBC-Perceived_Behavioural_Control has an indirect effect on EI.*

*H3 _f_: PCE-Perceived_Collective_Efficacy has an indirect effect on EI.*

It has been confirmed that knowledge and experience are decisive in identifying and exploiting business opportunities”. ^63^ ^,p.108^ Nevertheless, people with disabilities generally have lower levels of education and experience. ^31^^,^^48^^,^^64^ Consequently, this could affect the *perception of opportunities* and, therefore, EI. ^65^ Even so, because of the limited number of opportunities they have and the myriad barriers they must overcome, ^13^ people with disabilities are forced to tap into all their potential ^3^ and develop skills, personality traits, and creativity to seize opportunities to conduct new businesses in an innovative way. ^12^^,^^13^^,^^41^

Some studies confirm that certain types of disabilities allow a greater level of creativity, thus facilitating the identification of entrepreneurship opportunities. ^66^ Similarly, Wiklund *et al*. ^25^ state that people who suffer from attention deficit disorder tend to develop certain capabilities that allow them to identify market opportunities, which would ordinarily go unnoticed by others. This may also benefit the development of EI. Caldwell *et al*. ^12^ show how people with disabilities attempt to overcome and confront challenges and develop new ideas that seek to find answers and solutions to the problems they face. These circumstances favor social entrepreneurship.

There is also evidence that individuals who tend to adapt easily to new circumstances and barriers often have psychological resources, such as resilience, which is a trait associated with a successful entrepreneur. ^58^ RES-Resilience allows them to recognize business opportunities and mobilize resources to overcome the uncertainty of every business. In any case, the PO-Perception_of_Opportunities must be framed in terms of how desirable and feasible entrepreneurship is on a personal level. ^28^

Entrepreneurship is likely desirable for people with disabilities because of their limited employment options ^8^ and the stigmas and discrimination they face. ^12^^,^^22^^,^^26^ Considering what we have stated, we developed the following list of complementary hypotheses:

*H4 _a_ **:** PF-Perceived_Feasibility has a direct effect on PO-Perceived_Opportunities.*

*H4 _b_: PD-Perceived_Desirability has a direct effect on PO.*

*H4 _c_: PO has a direct effect on EI.*

### Resilience as a driver of entrepreneurial intentions in people with disabilities

RES-Resilience is the ability to remain relatively stable and healthy, either at a psychological or physical level, against the consequences of complex and painful events. ^67^ In the face of traumatic situations, RES-Resilience can cause emotions that can counteract the objective negativity that such circumstances could represent and produce positive experiences. ^68^

Although the concept of RES-Resilience has been systematically ignored in the literature that focuses on entrepreneurship, it resonates strongly, particularly when resilience is associated with EI. ^29^^,^^58^^,^^68^ Those who have studied the correlation often highlight the presence of a significant influence between RES-Resilience and EI. ^58^^,^^68^^,^^69^

It is almost a cliché to claim that people with disabilities tend to be resilient and persevere. ^13^ In fact, authors such as Wiklund *et al*. ^25^ confirm the above when they state that people with disabilities develop skills and resources that improve RES-Resilience, and that this circumstance in turn allows them to develop certain capabilities typical of entrepreneurial activity, such as self-confidence and a higher risk tolerance. ^7^^,^^13^ In short, this would reinforce a proactive A-Attitude_toward_Entrepreneurship, and a higher level of PBC-Perceived_Behavioural_Control. In addition, experience in overcoming obstacles can be successfully translated into a stronger work ethic and tenacity. ^25^ This will benefit both A-Attitude_toward_Entrepreneurship and PBC-Perceived_Behavioural_Control, resulting in an increased EI. ^58^^,^^70^ Similarly, resilient individuals are characterized as more optimistic and generate positive feelings, which foster adaptation and openness to social support. ^71^ Social support is important in adverse, threatening, and risky moments. ^72^ However, these circumstances are present when resilient people tend to succeed, ^13^ which only reinforces positive perceptions of support from family and friends (again increasing EI).

The literature reflects studies on the influence of RES-Resilience on EI in people without disabilities. ^29^^,^^69^ In the same way, we have compared studies that also focus on analysing the influence of RES-Resilience on situations of war or conflict. ^58^ However, we did not find any specific analysis that explicitly covers the relationship between RES-Resilience and EI in people with disabilities. Furthermore, this gap is interesting in the entrepreneurship space. ^25^

We developed the corresponding working hypotheses, under the following terms:

*H5 _a_: RES-Resilience has a direct effect on A-Attitude_toward_Entrepreneurship.*

*H5 _b_: RES has a direct effect on SN-Subjective_Norm.*

*H5 _c_: RES has a direct effect on PBC-Perceived_Behavioural_Control.*

*H5 _d_: RES has an indirect effect on EI.*

## Methods

### Ethical considerations

This study received ethical approval from the Committee of Ethics of Universidad Pontificia Comillas of Madrid on 11 ^th^ May 2022. No formal written consent was recorded; however, all the people who responded to the questionnaire were informed about the purpose of the study and how the data would be used. Answering or not answering the questionnaire was optional.

### Sample

In April and May 2020, empirical analysis was carried out through an online questionnaire (written in Spanish) given to people with disabilities between the ages of 16 and 65 years, residing in diverse regions of Spain. As requested by our funder the questionnaire was distributed to people with all types of disabilities without any restrictions. The configuration settings ensured that none of the participants answered the questionnaire twice. The study determined the minimum sample size by multiplying the number of highest arrows pointing to a particular endogenous construct by 10, as recommended by Barclay, Higgins and Thompson (1995H). ^73^ We have 9 constructs of which 8 are endogenous. The total number of arrows pointing to them directly is 13. This implies that a minimum sample size of 130 would be sufficient for our analysis. Also, Hair *et al.* ^74^ mentioned that, if G*power analysis is employed, 52 observations are needed to reach a statistical power (G*power) of 80% to detect R2 values of at least 0.25 (with a 5% probability of error). After waiting two months to obtain responses from people with disabilities and after the organizations that collaborated with us made several reminders to them, the total number of people who answered the online questionnaire was 240. Of these 5 were incomplete data, so they were eliminated from the database. Thus, taking into account that our sample is 235 people, it is believed that the size is adequate to apply the PLS tool and thus be able to test the hypotheses raised.

### Data collection

To collect the data, we were assisted by workers from the Once Foundation, through the Inserta program, a worker from the Ilunion Organization, and 16 other organizations. In the data collection process, an attempt was made to obtain a sample whose structure was adjusted to the profile of the population under study. For this reason, different collaborating organizations were chosen for data collection, in order to try to bias the sample as little as possible. The organizations that collaborated with us contacted potential participants by sending them an informative email. This email explained the purpose of the study, how the data would be treated and the importance of their participation. In the same way, the email sent by the organizations included the link to access the online questionnaire. To ensure anonymity in the questionnaire, we established the restriction of not being able to collect the e-mail addresses of the participants. Likewise, the data published were shown in aggregate form to avoid singling out attacks. People with intellectual disabilities, as well as people with visual impairments, had support staff provided by some of these organizations. This means, for example, that in the case of people with visual disabilities who did not have the means to read the questionnaire, a person from the organization was in charge of reading the questionnaire and writing down their answers. On the other hand, in the case of people with intellectual disabilities, if they did not understand any of the questions, the organizations were there to provide support and explain the concepts they did not understand.

### Measures

The items included in the questionnaire were taken from validated scales using a 7-point Likert scale (1 = strongly disagree; 7 = strongly agree). The questions employed are shown in the *Underlying data.* ^75^

The *dependent variable* was EI-Entrepreneurial_Intention. This was measured using the six-item scale developed by Liñán *et al*. ^52^

*Independent variables:* PD-Perceived_Desirability was measured by Shapero and Sokol’s ^36^ and Shook and Bratinau’s ^49^ scales; PF-Perceived_Feasibility was measured using Shook and Bratinau’s ^49^ and Krueger *et al*. ^38^ scales; A-Attitude_Towards_Entrepreneurship, PBC-Perceived_Behavioural_Control, and SN-Subjective_Norm were measured by Liñán *et al*. ^52^ scale. PCE-Perceived_Collective_Efficacy was measured using Chen’s ^76^ and Esfandiar *et al*.’s ^28^ scales. PO-Perceived_Opportunities were measured using the scale employed in the GEM ^77^ study. Finally, RES-Resilience was measured using Sinclair and Wallston’s ^78^ scale.

*Control variables:* To avoid possible biases, we used the following control variables. ^28^^,^^34^^,^^53^ Age was coded as a categorical variable (16-29 years old = 1; 30-44 years = 2; 45-65 years = 3). Gender: coded as a dichotomous variable (Men = 1; Women = 2). EDU-Level_of_Education: is coded as a categorical variable (primary education = 1, secondary education = 2, higher level specific vocational training cycles = 3, university = 4, doctorate = 5, and others = 6). EE-Entrepreneurial_Experience: is coded as a dichotomous variable, where 1 = has entrepreneurial experience and 2 = does not have entrepreneurial experience.

## Analysis

Descriptive statistics were performed with SPSS 24 (IBM SPSS Statistics, RRID:SCR_016479). The proposed model was tested using Partial Least Squares-Structural Equation Modeling (PLS-SEM) with the SmartPLS 3.2.7 version, which has been widely used in entrepreneurship research. ^28^^,^^79^ In addition, it is considered appropriate for complex structural models. ^80^ The analysis is developed in two stages.

1. *Assessment of the measurement model:* a) This includes a reliability analysis of the indicators. As shown in Table 1, several indicators must be removed. (ACT=0.429; ACT5=0.611; PBC5=0.641; PBC6=0.500; EI5=0.256; EI6=0.560; PO1=0.641; RES=0.660). The remaining variables have a loading greater than 0.707 for their corresponding constructs ( Table 1); b) for internal consistency, all the constructs meet the strict criteria for Cronbach’s alpha (0.8/0.9), as well as the Dijkstra-Henseler (rho_A) indicator and composite reliability (0.7). Additionally, the constructs reach convergent validity as they exceed the threshold required for AVE (0.5) ( Table 1).
  c) To analyze discriminant validity, we employ a Heterotrait-Monotrait ratio of correlations (HTMT), where values above 0.90 indicate multicollinearity problems. ^81^ We observe that the correlation between PD-Perceived_Desirability and A-Attitude_Towards_Entrepreneurship is 0.929 and the correlation between PD-Perceived_Desirability and PF-Perceived_Feasibility is 0.918. Then, to relax multicollinearity problems ^80^ we remove the most correlated indicators PD3, PF2, and ACT3 ( Table 2). After this process, this criterion is established including the confidence intervals ( Table 2).

**Table 1.** Assessment results of the measurement model.

Constructs/Variables	Constructs/variables factors	Cronbach’s alpha	Rho_A	Loads	Compound reliability	Median variance extracted
A	A1	0.937	0.944	0.893	0.939	0.885
	A2			0.986		
PBC	PBC1	0.910	0.915	0.830	0.912	0.721
	PBC 2			0.865		
	PBC 3			0.922		
	PBC 4			0.773		
PD	PD1	0.964	0.964	0.979	0.964	0.930
	PD2			0.950		
PF	PF1	1.000	1.000	1.000	1.000	1.000
EI	EI1	0.908	0.916	0.797	0.911	0.774
	EI3			0.919		
	EI4			0.918		
PO	PO2	1.000	1.000	1.000	1.000	1.000
PCE	PCE1	0.955	0.955	0.942	0.955	0.876
	PCE2			0.951		
	PCE3			0.915		
RES	RES2	0.875	0.875	0.827	0.875	0.699
	RES3			0.834		
	RES4			0.847		
SN	SN1	0.926	0.929	0.869	0.927	0.810
	SN2			0.948		
	SN3			0.882		
Age (control)	Age	1.000	1.000	1.000	1.000	1.000
EE (control)	EE	1.000	1.000	1.000	1.000	1.000
Gender (control)	Gender	1.000	1.000	1.000	1.000	1.000
Edu (control)	Edu	1.000	1.000	1.000	1.000	1.000

A = Attitude_Towards_Entrepreneurship, PBC = Perceived_Behavioural_Control, PD = Perceived_Desirability, EE = Entrepreneurial_Experience, Edu = Level_of_Education, EI = Entrepreneurial_Intention, PO = Perceived_Opportunities, PCE = Perceived_Collective_Efficacy, RES = Resilience, SN = Subjective_Norms, PF = Perceived_Feasibility.

**Table 2.** Discriminant validity criteria: Heterotrait-Monotrait ratio (HTMT).

	A	PBC	PCE	EI	SN	PO	PD	PF	RES
**A**	**-**	-	-	-	-	-	-	-	-
**PBC**	0.643	-	-	-	-	-	-	-	-
**PCE**	0.449	0.593	-	-	-	-	-	-	-
**EI**	0.812	0.806	0.517	-	-	-	-	-	-
**SN**	0.522	0.494	0.541	0.612	-	-	-	-	-
**PO**	0.590	0.707	0.537	0.727	0.415	-	-	-	-
**PD**	0.811	0.722	0.538	0.836	0.589	0.686	-	-	-
**PF**	0.764	0.717	0.528	0.828	0.566	0.660	0.875	-	-
**RES**	0.422	0.694	0.589	0.535	0.588	0.582	0.562	0.548	**-**

A = Attitude_Towards_Entrepreneurship, PBC = Perceived_Behavioural_Control, PD = Perceived_Desirability, EE = Entrepreneurial_Experience, Edu = Level_of_Education, EI = Entrepreneurial_Intention, PO = Perceived_Opportunities, PCE = Perceived_Collective_Efficacy, RES = Resilience, SN = Subjective_Norms, PF = Perceived_Feasibility.

First, the possible collinearity of the models is rejected based on the Variance Inflaction Factor (VIF) value, ^74^ except for the relationship between PD-Perceived_Desirability and EI, which is 0.508 when the limit is 0.500 ( Table 3).

**Figure 3.**
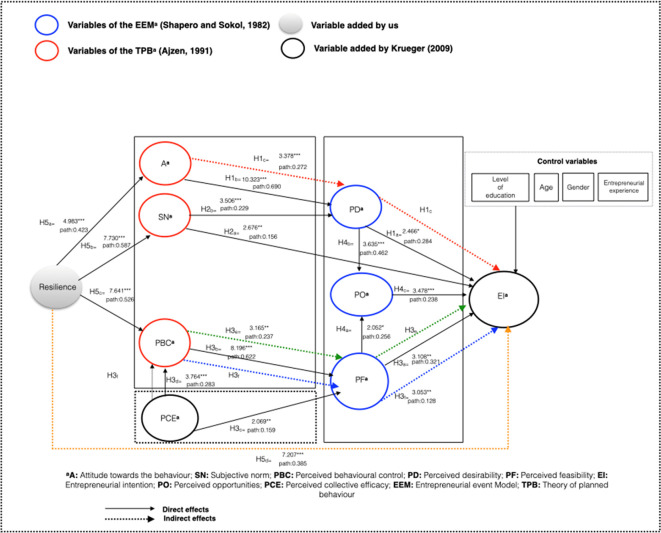
Final Structural model results. This figure shows the results obtained in each of the hypotheses raised. Resilience does have a direct and significant influence on attitude, subjective_norm, and perceived_behavioral_control (H5a; H5b; H5c_Accepted), and indirectly on EI (H5d_Accepted). Attitude directly and significantly influences perceived_desirability (H1b_Accepted) and indirectly EI (H1c_Accepted). Subjective_norm directly affects perceived_desirability and EI significantly (H2b;H2a_Accepted). Also, perceived_behavioral_control affects significantly and directly perceived_feasibility (H3b_Accepted) and indirectly EI (H2e_Accepted). Perceived_collective_efficacy affects directly and significantly perceived_behavioral_control (H3d_Accepted) and indirectly EI (H3f_Accepted). Perceived_desirability directly and significantly affects EI (H1a_Accepted) and perceived opportunities (H4b_Accepted). Perceived_feasibility significantly affects EI and perceived_opportunities (H3a:H4a_Accepted). Perceived_ opportunities directly and significantly influences EI (H4c_Accepted).

2. *In the study of the structural relationships among the constructs (once the first stage is accomplished) we have used*: a) The coefficient of determination (R ^2^), where R ^2^ exceeds the required values (ACT=0.179; PBC=0.531; PF=0.530; EI=0.791; SN=0.345; PO=0.486; PD=0.693) in all cases; b) The Stone-Geisser’s Q ^2^ ^82^^,^^83^ to check the model’s capability to predict. We use the cross-validity redundancy to estimate the predictive relevance of our model ^84^ obtaining a Q ^2^ of 0.596 (See Figure 2) which is an indication of a highly predictive model; ^74^ and c) the model fit, using the standardized root mean square residual (SRMR) and the normed fit index (NFI). SRMR fit values of 0 would indicate perfect fit and less than 0.05, acceptable fit. NFI values above 0.90 are considered acceptable. ^85^ Our SRMR value is 0.030, and the NFI value is 0.918, indicating that our model is well specified. Consistent bootstrapping (5,000 samples) is used to generate standard errors and t-statistics, which allow us to verify our hypothesis. ^84^ Figure 3 shows the results of the model.

**Table 3.** Collinearity Variance Inflaction Factor (VIFs).

	A	PBC	PCE	EI	SN	PO	PD	PF	RES
A	-	-	-	-	-	-	1.373		-
PBC	-	-	-	-	-	-	-	1.541	-
PCE	-	1.532	-	-	-	-	-	1.541	-
EI	-	-	-	-	-	-	-	-	-
SN	-	-	-	1.577	-	-	1.373	-	-
PO	-	-	-	2.055	-	-	-	-	-
PD	-	-	-	5.080	-	4.264	-	-	-
PF	-	-	-	4.544	-	4.264	-	-	-
RES	1.000	1.532	-	-	1.000	-	-	-	-

A = Attitude_Towards_Entrepreneurship, PBC = Perceived_Behavioural_Control, PD = Perceived_Desirability, EE = Entrepreneurial_Experience, Edu = Level_of_Education, EI = Entrepreneurial_Intention, PO = Perceived_Opportunities, PCE = Perceived_Collective_Efficacy, RES = Resilience, SN = Subjective_Norms, PF = Perceived_Feasibility.

## Results and discussion

The total number of people who answered the online questionnaire was 240. Of these 5 were incomplete data, so they were eliminated from the database. The final sample comprised 235 respondents (descriptive statistics in Table 4 performed with SPSS 24). ^75^

**Table 4.** Sample statistics.

Description	N=235	%	Description	N=235	%
**Gender**			**Type of disability**		
Men	148	62.09%	Auditive	9	3.83%
Women	87	37.02%	Auditive + Physical	6	2.55%
**TOTAL**	**235**	**100%**	Auditive + Physical + Psychosocial	1	0.43%
**Age**			Auditive + Physical + Visual	1	0.43%
16-29 years old	35	14.89%	Visual	21	8.94%
30-44 years old	100	42.55%	Physical	129	54.89%
45-65 years old	100	42.55%	Physical + Cognitive	7	2.98%
**TOTAL**	**235**	**100%**	Physical + Visual	11	4.68%
**Marital status**			Cognitive	17	7.23%
Single	124	52.77%	Psychosocial	5	2.13%
Married	73	31.06%	Physical and mental	2	0.85%
Divorced	35	14.89%	Mental	4	1.70%
Widowed	1	0.43%	Cognitive and auditive	2	0.85%
Other	2	0.85%	Mental and sensitive	2	0.85%
**TOTAL**	**235**	**100%**	Sensitive	1	0.43%
**Level of education**			Others	18	7.66%
Primary	42	17.87%	**TOTAL**	**235**	**100%**
Middle School	32	13.62%			
Professional training	70	29.79%	**Disability degree**		
College	70	29.79%	Moderate disability	143	60.85%
PhD	4	1.70%	Severe disability	54	22.98%
Other	17	7.23%	Very severe disability	38	16.17%
**TOTAL**	**235**	**100%**	**TOTAL**	**235**	**100%**

We assert that the PD-Perceived_Desirability has a direct influence over EI (path: 0.284, t-value: 2.466); A_Attitude_Toward_Entrepreneurship increases PD-Perceived_Desirability (path: 0.690, t-value: 10.323), and A_Attitude towards the fact that becoming an entrepreneur has an influence on EI through the mediation of PD-Perceived_Desirability ( *path* 0.272, t-value: 3.378). Thus, we accept hypotheses H1 _a_, H1 _b,_ and H1 _c._

These results have been obtained by other authors in prior studies of people without disabilities ^28^^,^^49^^,^^54^; however, these are considered novel in the study of entrepreneurship among people with disabilities. The importance of shaping positive A_Attitude_Toward_Entrepreneurship can be observed, because they are led to perceive entrepreneurship as a more desirable career choice. ^49^ This has already been established by others, ^36^ who show that people with a high level of PD-Perceived_Desirability would have a greater probability of developing EI. Governments and public administrations, as well as educational institutions, non-profit organizations and vocational rehabilitation centers, specialist and educators, could attempt to stimulate entrepreneurship in people with disabilities, so that they might perceive it as a feasible employment option. Doing so requires a process of cultural change, because Spanish culture does not favor entrepreneurship. ^63^ However, culture has a strong influence on the PD-Perceived_Desirability of entrepreneurship. ^36^

Similarly, it is necessary to create positive A_Attitude_Toward_Entrepreneurship among people with disabilities by showing them and their influential loved-ones the possible results on a personal or professional level. ^42^ Therefore, the key is to send a clear message to loved ones, who could break down barriers and significantly influence a person with disabilities’ interest and success in entrepreneurship. ^22^^,^^48^ Such positive encouragement could lead to the freedom and economic independence they seek. ^5^^,^^16^ In this regards the vocational rehabilitation centers and specialist play a key role.

SN-Subjective_Norm has a direct and significant influence on the EI of people with disabilities ( *path:* 0.156; t-value: 2.676) as does PD-Perceived_Desirability ( *path:* 0.229; t-value: 3.506). This leads us to accept H2 _a_ and H2 _b_. We tested the importance of the opinions of family members, friends, and colleagues for people with disabilities when deciding to become an entrepreneur. ^6^ This finding concurs with Martínez-León *et al*., ^42^ who showed that for people with disabilities who wanted to become entrepreneurs, moral support from one’s immediate circle was even more important than having start-up funding. Therefore, the opinions of family members, friends, and other close relatives had great relevance in decision-making. In fact, Shen *et al*. ^39^ showed that college students who depended on their parents, emotionally and economically, tended to develop a higher EI.

Regarding the positive and significant relationship found between the SN-Subjective_Norm and PD-Perceived_Desirability, this relationship has been supported by other studies on people without disabilities. ^54^ However, this did not seem to be a trend in people without disabilities, especially in cultures that have a strong sense of individualism. ^28^^,^^53^ Nonetheless, it makes sense in our study. Thus, it is advisable that rehabilitation centers, educators and specialist focus on family members and friends who can provide critical encouragement.

PF-Perceived_Feasibility has a significant influence on EI ( *path*: 0.321; t-value: 3.108), leading us to accept H3 _a_. People with disabilities who perceive that they are capable of becoming entrepreneurs develop a higher level of EI. This is in line with other studies on people without disabilities. ^28^^,^^39^^,^^59^ Similarly, observations show that PBC-Perceived_Behavioural_Control has an influence on PF-Perceived_Feasibility, with the acceptance of H3 _b_ ( *Path:* 0.622; t-value: 8.196), which goes hand in hand with studies on people without disabilities. ^27^^,^^28^^,^^49^ Therefore, showing that those individuals who perceive that they have more control over their own skills feel that it is more feasible to become an entrepreneur. Thus, it becomes evident that the relationship between PBC-Perceived_Behavioural_Control and PF-Perceived_Feasibility is direct, positive, and significant. ^27^^,^^49^

By contrast, we observed that the PCE-Perceived_Collective_Efficacy has a direct and significant influence on PF-Perceived_Feasibility, which leads us to accept H3 _c_ ( *Path*: 0.159; t-value: 2.069), consistent with other studies about people without disabilities. ^28^ Therefore, it seems evident that for people with disabilities, starting a business with other partners or in a collective setting is critical, because social support from their environment is very important. ^42^ Support can come from close relatives or people who Granovetter ^61^ refers to as having weak relationships, but who may provide the most current resources and knowledge. This support increases the feeling that they can become entrepreneurs, which reflects PF-Perceived_Feasibility. ^28^

In addition, the PCE-Perceived_Collective_Efficacy increases PBC-Perceived_Behavioural_Control on behalf of the individual considering the entrepreneur, who can rely on support and help provided by others. ^62^ Therefore, we accept H3 _d_ ( *path*: 0.283; t-value:3.764). In fact, when a person with disabilities increases their PBC-Perceived_Behavioural_Control, they increase their PF-Perceived_Feasibility *,* which results in a higher EI. Finally, these considerations allow us to accept both hypothesis H3 _e_ ( *Path*: 0.237; t-value: 3.165) and hypothesis H3 _f_ ( *Path*: 0,128; t-value: 3.053) as true.

Thus, in our context, we confirm how important it is for people with disabilities to start a business in cooperation with others and not individually. ^28^ Some support can benefit the process of overcoming existing barriers, as well as the growth and development of new networks, which increases the individuals’ levels of PBC-Perceived_Behavioural_Control. Additionally, access to capital, resources, and high-quality information increases with collaboration, without disregarding the importance of moral support in collaborative relationships. ^13^^,^^42^ This ultimately results in an increase in *the* PF-Perceived_Feasibility and EI. Hence, we confirm what is stated in Pérez-Macías and Fernández-Fernández, ^2^ where many participants talked about the need for partnership in entrepreneurship, which could make them feel safer and more supported.

Whereas it seems that when someone considers themselves capable of performing an entrepreneurial action ( *perceived feasibility*), the PO-Perception_of_Opportunities increases. This leads us to accept H4 _a_ (path: 0.256; t-value: 2.052), which aligns with the findings of other studies. ^28^ When people with disabilities have knowledge, they often consider themselves capable of becoming entrepreneurs and have high EI. To fill any gaps in education and experience, providing specialized training is important, ^86^ and an influential mentor could help with certain processes and perceptions. ^50^

In the same way, considering entrepreneurship desirable helps increase the PO-Perception_of_Opportunities leading to the acceptance of H4 _b_ ( *path*: 0.462; t-value: 3.635), which aligns with Esfandiar *et al*. ^28^ Additionally, the PO-Perception_of_Opportunities increases the EI of people with disabilities ( *path*: 0.238; t-value: 3.478). This leads to accept H4 _c_. ^28^^,^^65^

Taking into consideration the above, specialized training and mentors or role models are essential to help people with disabilities feel safer about becoming entrepreneurs. These support systems could help people with disabilities feel a greater desire to become entrepreneurs and allow them to detect a greater number of entrepreneurial opportunities and to develop a higher EI. This could be either because of the confidence derived from the ad hoc training received, or from the positive life examples of other entrepreneurs. This could help alleviate negative experiences and discrimination. ^12^^,^^22^

Likewise, we proved that when an individual is more resilient, it allows them to have more favourable A-Attitudes_Toward_Entrepreneurship ( *path*: 0.423; t-value: 4.983). Thus, we confirm what other authors stated previously: resilient individuals tend to develop more favorable A-Attitudes_Toward_Entrepreneurship and, therefore, have a higher EI. ^29^^,^^58^^,^^68^^,^^69^ Renko, Bullough, and Saeed ^68^ point out the key role of RES-Resilience in entrepreneurship, leading us to accept H5 _a_ and H5 _d_, where RES-Resilience has an indirect influence on EI ( *path*: 0.385; t-value: 7.207).

We also confirm that RES-Resilience has a positive influence on the SN-Subjective_Norm ( *path*: 0.587; t-value: 7.730), which leads us to accept H5 _b_, meaning that the more resilient an individual is, the more positive their A-Attitudes_Toward_Entrepreneurship. ^71^ This positive A-Attitudes_Toward_Entrepreneurship and coping ability are perceived positively by close-loved ones, who promote even greater support. ^71^

Finally, we confirm that RES-Resilience has a direct and significant influence on PBC-Perceived_Behavioural_Control *,* which leads us to accept H5 _c_ ( *path*: 0.526; t-value: 7.641). This makes sense because challenging environments trigger RES-Resilience; in the case of people with disabilities, RES-Resilience enables a more determined will to become an entrepreneur because individuals feel better prepared and more likely to succeed. ^7^^,^^13^ Among the distinguishing traits developed in challenging environments, it is worth highlighting the different barriers faced by people with disabilities, ^7^^,^^13^^,^^22^ fewer work opportunities, ^8^ and the stigmatization and discrimination they. ^12^^,^^22^^,^^26^ RES-Resilience improves positive *attitudes* and *self-confidence.* ^58^

It is important to emphasize that RES-Resilience plays a critical role that must be considered, especially in adverse environments in which people with disabilities live. Consequently, it would be advisable to carry out training activities that work on said skill in the form of a simulation, which may be a practical methodology (see Table 5 for a summary).

**Table 5.** Results of hypothesis testing.

H	Direct association	Original sample	Sample mean	Standard deviation	T tests	P values
H1 _a_	PD->EI	0.284	0.289	0.115	2.466 ^*^	0.014
H1 _b_	A->PD	0.690	0.690	0.067	10.323 ^***^	0.000
H1 _c_	A->EI ^indirect^	0.272	0.276	0.081	3.378 ^***^	0.001
H2 _a_	SN->EI	0.156	0.153	0.058	2.676 ^**^	0.007
H2 _b_	SN->PD	0.229	0.230	0.065	3.506 ^***^	0.000
H3 _a_	PF->EI	0.321	0.318	0.103	3.108 ^**^	0.002
H3 _b_	PBC->PF	0.622	0.623	0.076	8.196 ^***^	0.000
H3 _c_	PCE->PF	0.159	0.158	0.077	2.069 ^*^	0.039
H3 _d_	PCE->PBC	0.283	0.285	0.075	3.764 ^***^	0.000
H3 _e_	PBC->EI ^indirect^	0.237	0.238	0.075	3.165 ^**^	0.002
H3 _f_	PCE->EI ^indirect^	0.128	0.127	0.042	3.053 ^**^	0.002
H4 _a_	PF->PO	0.256	0.262	0.125	2.052 ^*^	0.040
H4 _b_	PD->PO	0.462	0.455	0.127	3.635 ^***^	0.000
H4 _c_	PO->EI	0.238	0.239	0.068	3.478 ^***^	0.000
H5 _a_	RES->A	0.423	0.423	0.085	4.983 ^***^	0.000
H5 _b_	RES->SN	0.587	0.585	0.076	7.730 ^***^	0.000
H5 _c_	RES->PBC	0.526	0.524	0.069	7.641 ^***^	0.000
H5 _d_	RES->EI ^indirect^	0.385	0.385	0.060	7.207 ^***^	0.000

A = Attitude_Towards_Entrepreneurship, PBC = Perceived_Behavioural_Control, PD = Perceived_Desirability, EE = Entrepreneurial_Experience, Edu = Level_of_Education, EI = Entrepreneurial_Intention, PO = Perceived_Opportunities, PCE = Perceived_Collective_Efficacy, RES = Resilience, SN = Subjective_Norms, PF= Perceived_Feasibility.

Note: *p < 0.05; **p < 0.01; ***p < 0.001; based on a one-tailed Student’s t-distribution (499): t(0.05; 499) = 1.6479, t(0.01; 499) = 2.3338, t(0.001; 499) = 3.1066.

## Conclusions

Among the 17 Sustainable Development Goals (SDGs), the 2030 Schedule defines (among others) to “reduce inequalities within and among countries” and to “promote peaceful and inclusive societies for sustainable development, provide access to justice for all and building effective, accountable and inclusive institutions at all levels.” ^[^ ^1^ ^]^

As part of making progress towards achieving these goals, this work aims to deepen the knowledge of the variables that determine the EI of people with disabilities, based on the theory-based contributions of Krueger ^27^ and Esfandiar *et al*., ^28^ and including the RES-Resilience construct. ^2^

Therefore, the aim is to provide an understanding of the subject of EI among people with disabilities to help reduce the gap in employment between people with disabilities and those without and to improve the regional development of Spain. We aim to promote a form of occupational activity based more on creating employment and self-employment, rather than on the search for salaried work. The ultimate goal of this study is to leverage systematic academic research to achieve a more inclusive society, which also reduces the social costs of unemployment benefits and subsistence subsidies for people with disabilities. Ultimately, this study aims to contribute to improving the self-esteem and quality of life of people with disabilities through its contribution to society.

The results attest to the importance of personalized factors in the entrepreneurial process. The model we use shows the importance of positive A-Attitudes_Toward_Entrepreneurship, PD-Perceived_Desirability, and PF-Perceived_Feasibility. In other words, the people surveyed believe that they are capable of entrepreneurship. PBC-Perceived_Behavioural_Control, PO-Perception_of_Opportunities, and PCE-Perceived_Collective_Efficacy should also be presented as relevant aspects in the entrepreneurial process. This is something that had already been found in other studies carried out among people without disabilities, but which, so far, we have not seen corroborated in analyses of people with disabilities. Given the importance of support and companionship for people with disabilities, both at the beginning and in the process of the entrepreneurial journey, it is advisable to create a technological platform for entrepreneurs, with and without disabilities, to establish mutual interconnection and to share mutual motivations, concerns, ideas, experiences, best practices, objectives, and so on. The reason for creating an App is that people with disabilities face many barriers, such as mobility. ^2^ Therefore, the App would greatly facilitate interaction between people with disabilities and people without disabilities without the need to move from home. However, it is necessary that the people who work with them in rehabilitation centers, for example, have knowledge of the advantages of entrepreneurship and know the existing tools (for example, the App), in order to be able to inform people with disabilities.

Similarly, and unlike studies carried out with people without disabilities, where the variable SN - Subjective_Norm creates controversy, in the specific case of people with disabilities, we observe the importance of other people's opinions and the way in which this perception can influence the EI of differently abled people directly and significantly. Then, it is necessary to educate family members, friends, and relatives of such people, to make them aware of the importance of their opinions in terms of decision-making about entrepreneurship and employment. In these sense, rehabilitation centers, rehabilitation specialist and educators play a key role.

Similarly, the RES-Resilience variable, which is beginning to emerge frequently among studies on entrepreneurship, is seen to be important to the EI of people with disabilities. We know that persistence, perseverance, strength, and a future-focused orientation are crucial aspects in any entrepreneurial process; they are even more significant in the specific case of people with disabilities. Then, considering the importance of personal *attitudes*, the fact that the person feels capable of carrying out certain actions and *resilience*, we recommend the establishment of coaching groups aimed at strengthening the personality traits and character resources that we know can help and encourage entrepreneurship. For this purpose, it would be convenient to alert not only educational centers and rehabilitation centers, but also the abundant list of organizations dedicated to the design and implementation of tools that facilitate social inclusion through entrepreneurship.

Finally, entrepreneurial culture in Spain is not very popular. However, well-thought-out messages, elaborated with art and communicated effectively, could contribute to more people taking entrepreneurship seriously, as an alternative to traditional employment. Consequently, we encourage that entrepreneurship be branded as a lever of social progress and personal development for all people. We believe that well-designed and deployed campaigns could emphasize the value –in quantitative terms–that entrepreneurship brings to owners and to various stakeholders, interest groups, and agents, as well as society.

This study has several limitations. First, it was conducted in Spain. It would be interesting to develop this study in other countries to determine if cultural factors change the prediction capacity of this model. Additionally, generalizations should be made with care. This is because it may be applicable to countries such as Spain with a low entrepreneurial culture, but the same results may not be obtained in more entrepreneurial countries. In addition, in the VIF matrix, the correlation between PD and EI was 0.5080, above the limit (0.5).

Considering that we live in the information age, when information provides power and competitive advantage, it would be useful to develop an unsupervised machine learning model. Considering that machine learning models learn from the data provided, such a model can create segmented groups by disabilities. This would allow us to classify people with similar disabilities in the same dataset and differentiate between different characteristics in different sets. This would help researchers distinguish between people with entrepreneurial tendencies and those with no entrepreneurial spirit. Finally, it would be interesting to add other personal variables that can help us better understand this population and decipher what drives their entrepreneurial intentions. Likewise, it will be interesting to consider self-efficacy as a multidimensional construct considering its importance is several contexts. ^59^

## Data availability

### Underlying data

Figshare: People with disabilities Dataset in spanish. https://doi.org/10.6084/m9.figshare.19615710.v2. ^75^

Data are available under the terms of the Creative Commons Attribution 4.0 International license (CC-BY 4.0).

## Footnotes

^1^

https://sdgs.un.org/goals
