# Supplementary material for: Resilience and entrepreneurial intentions of people with disabilities: in search of the Sustainable Development Goals (SDGs)
Source: F1000Res. 2022 Jul 1;11:726. [Version 1] doi: 10.12688/f1000research.113565.1 (PMC9475206; doi:10.12688/f1000research.113565.1)
Supplement: Peer review history

# Peer review: Reviewer response for version 1

**DOI:** 10.5256/f1000research.125298.r147748

**Published:** 2022-09-14

**Authors:** Yuleinys A Castillo

**Affiliations:** 1 California State University,, Fresno, CA, USA

This novel topic will be of interest to the readers of the journal especially considering the importance of resilience and entrepreneurial intentions of people with disabilities.   

**Abstract: **This is a good attempt to summarize the manuscript. You can provide context to this study since the SDG can be confusing for some readers. Revise your organization to better connect ideas.

**General**

- Properly cite your statements.
- There are numerous minor editorial errors throughout the manuscript (e.g., plurals, hyphens, possessive punctuation)
- Properly format references list
- Revise for the flow of ideas, including sentence structure and proper tense used in writing.

**Body**

- Overall, the introduction is well organized. However, consider enhancing transitions between major sections and reorganizing paragraphs.
- What’s ODISMET?
- If possible, add or modify information to understand the context of this study
- Expand your section on your framework or aim to explain it in a simple manner.
- Combine your paragraphs. Some of your paragraphs are one sentence only.

**Method**

- Revisit to improve readability
- Any analysis for demographics?

**Discussion**

- Reorganize information to support your arguments
- Any other implications
- Make sure that you organize information together to improve readability
- Revise some of your sentences, as they seem like incomplete ideas.  

Is the work clearly and accurately presented and does it cite the current literature?

Yes

If applicable, is the statistical analysis and its interpretation appropriate?

Yes

Are all the source data underlying the results available to ensure full reproducibility?

Yes

Is the study design appropriate and is the work technically sound?

Yes

Are the conclusions drawn adequately supported by the results?

Yes

Are sufficient details of methods and analysis provided to allow replication by others?

Yes

Reviewer Expertise:

disability studies, vocational services, mental health

I confirm that I have read this submission and believe that I have an appropriate level of expertise to confirm that it is of an acceptable scientific standard.

# Peer review: Reviewer response for version 1

**DOI:** 10.5256/f1000research.125298.r146532

**Published:** 2022-08-31

**Authors:** Virginia Barba-Sanchez

**Affiliations:** 1 ENSITMA R.G., Department of Business Science, Universidad de Castilla-La Mancha, Albacete, Spain

I appreciate the authors for concentrating on such a relevant issue. I enjoyed reading the manuscript and found it to be an informative and intriguing study on the variables that determine the entrepreneurial intention of people with disabilities. The general quality of the manuscript is so good. Nevertheless, certain issues are inherent in this manuscript, and I propose a minor revision although the article might be accepted for indexing as is. Therefore, I've made the following observations and suggestions to improve the quality of the current work:

1. The manuscript's title should be catchy and reader-friendly. I suggest that the authors reconsider it.
2. The 2030 Agenda for Sustainable Development of the United Nations (UN) includes in its Sustainable Development Goal (SDG) 8.5 “to achieve full and productive employment and decent work for all women and men, including for young people and persons with disabilities (PWD), and equal pay for work of equal value” (p. 22). For their part, the Entities of the Social and Solidarity Economy (SSE), by their very nature, are presumed to be more sensitive in the labour insertion of groups in danger of social exclusion than are the Capitalist companies (CC) such as people with disabilities (PwD). Nevertheless, Calderón et al. (2021) provide empirical evidence of the difficult integration of PwD even in SSE. Therefore, I suggest using this reference to enrich the justification for your manuscript:
*Calderón, M.J., Calderón, B, Barba-Sánchez, V. (2020). Labour Inclusion of People with Disabilities: What Role Do the Social and Solidarity Economy Entities Play?* ^1^
3. You stated: “The study determined the minimum sample size by multiplying the number of highest arrows pointing to a particular endogenous construct by 10, as recommended by Barclay, Higgins, and Thompson (1995H).” I agree that the sample size depends on the complexity of the model, but the use of G*power is correct and appropriate for the assessed sample size. Why use too the rule of Barclay, Higgins, and Thompson (1995)? Please remove this last one.

Good luck with your future work.

Is the work clearly and accurately presented and does it cite the current literature?

Yes

If applicable, is the statistical analysis and its interpretation appropriate?

Yes

Are all the source data underlying the results available to ensure full reproducibility?

Yes

Is the study design appropriate and is the work technically sound?

Yes

Are the conclusions drawn adequately supported by the results?

Yes

Are sufficient details of methods and analysis provided to allow replication by others?

Yes

Reviewer Expertise:

Entrepreneurship, Entrepreneur with Disabilities, Social value, ICT

I confirm that I have read this submission and believe that I have an appropriate level of expertise to confirm that it is of an acceptable scientific standard.

## References

[1] : Labour Inclusion of People with Disabilities: What Role Do the Social and Solidarity Economy Entities Play?. *Sustainability* .2020;12(3) : 10.3390/su12031079 10.3390/su12031079
